# Spatial distribution of intimate partner violence in Cambodia: results from the 2021–22 Demographic and Health Survey

**DOI:** 10.3389/fpubh.2025.1509076

**Published:** 2025-05-09

**Authors:** Masood Ali Shaikh

**Affiliations:** College of Medicine, Korea University, Seoul, Republic of Korea

**Keywords:** intimate partner violence, women, spatial analysis, hotspot analysis, Cambodia

## Abstract

**Background:**

Male-perpetrated Intimate Partner Violence (IPV) against women is recognized as a global public health and human rights issue. In Cambodia, the spatial aspects of IPV have not previously been explored.

**Methods:**

Using data from the latest Demographic and Health Survey (2021–22), an analysis of IPV’s spatial distribution was conducted.

**Results:**

The analysis revealed clustering, with a Global Moran’s I Index of 0.223079 and a statistically highly significant *p*-value of <0.0001. Distinct hot and cold spots of IPV prevalence emerged, supported by kriging analysis. The southeastern part of Mondul Kiri province exhibited the highest IPV proportions, ranging from over 50 to 100%, while the western parts of Koh Kong and Preah Sihanouk provinces had the lowest IPV proportions, ranging from 0 to 5%. Large areas in the northern half of the country, as well as smaller regions in the southern provinces of Kampot, Takeo, Kandal, and Prey Veng, showed IPV prevalence proportions ranging from greater than 25 to 50%. In contrast, the lower half of the country had comparatively lower IPV prevalence, with proportions between greater than 5 and 25%.

**Conclusion:**

The observed spatial clustering of IPV and the identification of high-prevalence areas, underscore the importance of incorporating spatial analysis into IPV research. These findings can guide geographically targeted public health policies and health education programs aimed at mitigating IPV in the most affected regions.

## Introduction

Intimate Partner Violence (IPV) remains a pervasive public health challenge, significantly impacting the well-being and socio-economic development of societies globally. Defined as any behavior within an intimate relationship that causes physical, sexual, or psychological harm to those in the relationship (1); IPV is a violation of human rights and a major obstacle to achieving gender equality (1). Several studies indicate that the geographic distribution of IPV is not uniform, with certain areas within countries experiencing higher prevalence rates than others (2–5). This suggests that a localized approach, tailored to specific regions within countries, could potentially be more impactful in addressing IPV.

Cambodia is a Southeast Asian country bordered by Thailand, Laos, and Vietnam. In Cambodia, the issue of IPV has garnered increasing attention over recent years, given its complex interplay with socio-cultural, economic, and spatial factors. Despite significant strides in socio-economic development, Cambodia continues to face high rates of IPV, which is often intertwined with historical, cultural, and geographic dimensions. Cambodia, with its unique socio-political history, faces distinctive challenges in addressing IPV. The legacies of the Khmer Rouge regime, which devastated the social and cultural fabric of the country, continue to influence family structures and gender dynamics (6). Cambodian society is characterized by deeply rooted gender norms, which often contribute to the perpetuation of IPV. Women in Cambodia are often expected to conform to traditional roles of subservience and obedience to their male partners. Such socio-cultural expectations, coupled with limited economic opportunities for women, contribute to the vulnerability of women to IPV (7). While socio-cultural and economic determinants of IPV are well-documented in the literature, there is growing recognition that the spatial dimensions of IPV also play a crucial role in shaping risk patterns (2–5, 8, 9). These studies have demonstrated the importance of understanding IPV through a spatial lens, and have highlighted the geographic clustering of IPV and the role of regional factors in shaping IPV patterns.

Spatial analysis has become a powerful tool in public health research, enabling researchers to identify patterns and determinants of health outcomes across different geographic areas. By integrating Geographic Information Systems (GIS) technology with statistical analysis, spatial analysis can reveal the geographic clustering of IPV, identify socio-environmental correlates, and inform place-based interventions (9, 10).

The 2021–22 Cambodia Demographic and Health Survey estimated that one in five (20.70%) women experienced some form of IPV during their lifetime, perpetrated by their current or most recent husband or intimate partner (11). Although this statistic provides insight into the prevalence of IPV, the geographic distribution of IPV across Cambodia remains insufficiently explored, limiting the capacity of policymakers to design location-specific interventions. The spatial analysis of IPV in Cambodia offers an essential avenue for understanding the geographic distribution of this violence and informing targeted interventions to mitigate its impact.

The Demographic and Health Surveys are often the only nationally and subnational representative surveys in several developing countries, providing health and demographic indices including domestic violence and intimate partner violence (12). There are no studies on the spatial distribution of IPV in Cambodia. Studies elucidating the ‘place’ based estimates of IPV are imperative in Cambodia for better understanding and informing public health and, medical policies, in addition to health education and promotion endeavors addressing the socio-cultural norms underpinning and perpetuating such practices for eliminating this health and human rights menace. The primary objective of this study was to analyze the spatial distribution of IPV in Cambodia and to identify geographic hotspots where women were at greater risk of experiencing IPV; using the most recent Cambodia Demographic and Health Survey, conducted in 2021–22. By using spatial analytical techniques, this study aimed to uncover the underlying spatial patterns of IPV and examine the relationship between geographic location and IPV prevalence. Specifically, the study aimed to map the geographic distribution of IPV prevalence across Cambodia, and to identify spatial clusters or hotspots of IPV. Through these objectives, the study contributes to a deeper understanding of the spatial distribution of IPV in Cambodia, providing insights that can be crucial for developing targeted and effective interventions.

## Methods

This section describes the data sources, study area, and methods used to conduct a spatial analysis of IPV in Cambodia. The spatial analysis aimed to identify the geographic distribution, clustering, and hotspots of IPV prevalence using multiple geospatial and statistical techniques.

### Study area and data source

The anonymized Cambodia Demographic and Health Survey 2021–22 (CDHS2021-22) data were used for secondary analysis. The survey was implemented by the Cambodian National Institute of Statistics and the Ministry of Health, with technical assistance provided by ICF though The DHS program.

The 2019 General Population Census (GPC) of Cambodia served as the sampling framework for the CDHS 2021–22. The GPC includes a comprehensive list of all enumeration areas (EAs) in Cambodia, which were used as census counting units. For the CDHS 2021–22, a two-stage sampling design was implemented to generate estimates at the national, urban, rural, and provincial levels across Cambodia’s 25 provinces. In the first stage, 709 EAs or clusters were selected. The geographic point coordinates (latitude and longitude) for each EA were collected. To protect respondent confidentiality, the GPS coordinates were randomly displaced. The displacement was applied as follows: urban EAs were shifted by up to 2 kilometers, while rural EAs are displaced by up to 5 kilometers, with 1% of rural EAs displaced by as much as 10 kilometers. Importantly, this displacement was restricted to ensure that all points remain within the boundaries of the country, the DHS survey region, and the admin2 area.

In the second stage, 30 households were systematically chosen from each EA. One woman in each household was randomly selected for the administration of the domestic violence module, which included IPV questions. The domestic violence module was administered only after securing verbal informed consent and ensuring privacy. Women aged 15–49 years “who have ever had a husband or other intimate partner” were asked about having ever experienced either emotional, physical, and/or sexual violence perpetrated by the current or most recent husband for ever-married women, or current or most recent intimate partner for never-married women.

The Cambodian National Institute of Statistics and the Ministry of Health, along with the ICF Institutional Review Board, provided ethical approval for the CDHS 2021–22. For the secondary analysis of fully anonymized CDHS 2021–22 survey data, the author received approval from The Measure DHS via an online request form. Since this analysis utilized anonymized data, no additional ethical approval was necessary. Detailed information regarding the CDHS 2021–22, including the survey instruments, methodology, sampling techniques, and weights generation, is publicly available on The Measure DHS website.

### Study variable

A binary composite variable, “IPV,” was created. It was coded as “1” if the respondent reported having ever experienced any form of emotional, physical, or sexual intimate partner violence perpetrated by their current or most recent husband or intimate partner; otherwise, it was coded as “0.” As previously described: “Emotional IPV was deemed positive if the respondent reported that her male intimate partner had either ever humiliated her, threatened to harm her, insulted her, or made her feel bad. Physical IPV was based on respondent’s affirmative responses to either having been ever pushed, shaken, thrown something at, slapped, arm twisted or hair pulled, punched with fist or something that could hurt, kicked, dragged, strangled, burned, or threated with a knife, gun, or any weapon by her male intimate partner. While sexual violence was computed as present if respondent had ever been either physically forced into unwanted sex, unwanted sexual acts, or to perform unwanted sexual acts” (11).

### Data analysis

This study employed a secondary analysis of anonymized data from the CDHS 2021–22, accessed from the Measure DHS website,^1^ after receiving approval. This study utilized STATA version 18 (Texas, USA) to calculate weighted IPV prevalence at the EA level in Cambodia. For mapping and spatial analysis, ESRI’s ArcMap version 10.8.1 was employed. The administrative boundaries for Cambodia were downloaded from the Spatial Data Repository of The Demographic and Health Surveys Program (13). All maps were created using the World Geodetic System 1984 (WGS 84) Geographic Coordinate System (GCS).

The EA-level weighted IPV proportions were calculated in Stata, copied to an Excel file, and subsequently joined with the geographic coordinates of each EA in ArcMap. For three EAs geographic coordinates were not available and were removed. The spatial distribution of EA-level IPV proportions, expressed as percentages, was displayed on the map.

A spatial autocorrelation analysis was performed using the ‘Global Moran’s I’ statistic to evaluate whether there was a clustering pattern in the proportions of IPV across Cambodia. This statistic measures the degree to which similar values are spatially clustered in a two-dimensional space. The analysis yields a Global Moran’s I value, Z-score, and *p*-value, which indicate whether the data exhibits clustering, randomness, or dispersion. A Moran’s I value close to 1 suggests clustering, near −1 indicates dispersion, and values around 0 imply randomness. A *p*-value of less than 0.05 indicates statistically significant clustering, also known as spatial autocorrelation. In this study, spatial relationships were conceptualized using two methods: the zone of indifference and inverse distance approaches. For both methods, Euclidean distance was employed to calculate spatial weights. The zone of indifference approach combines a fixed distance threshold, within which all neighbors are assigned equal weights, and a gradual decay in influence for locations outside the threshold. This approach accounts for both immediate neighbors and more distant spatial interactions. In contrast, the inverse distance method assigns spatial weights inversely proportional to the Euclidean distance, emphasizing closer neighbors while diminishing the influence of distant ones.

The Getis-Ord Gi* statistic is a localized metric used to analyze spatial clustering. It evaluates how high or low values are distributed by comparing each point with its neighboring points. The output includes z-scores and *p*-values, which help identify areas where high or low values are spatially clustered. This method specifically determines if a feature with a high value is surrounded by similarly high values (hot spot) or by low values (cold spot). An optimized hot spot analysis was performed, which optimizes the process and pinpoints statistically significant spatial clusters. The optimal fixed distance band for this statistic was determined based on the average distance to the thirty nearest neighbors.

The EAs, represented as points with geographic coordinates on the map, display IPV proportions only at those specific locations. For areas without EAs, the values were estimated by interpolating data from the existing EAs. Ordinary Kriging, a predictive technique, was employed to estimate IPV proportions across the entire study area. The Kriging analysis was performed using the Spatial Analyst Extension in ArcGIS, with the default settings of Ordinary Kriging and a spherical semivariogram model. The number of points used for interpolation was set to 12. The choice of the Ordinary Kriging method and the spherical semivariogram model was based on their wide applicability and effectiveness in capturing spatial patterns in similar contexts.

## Results

**Figure 1:**
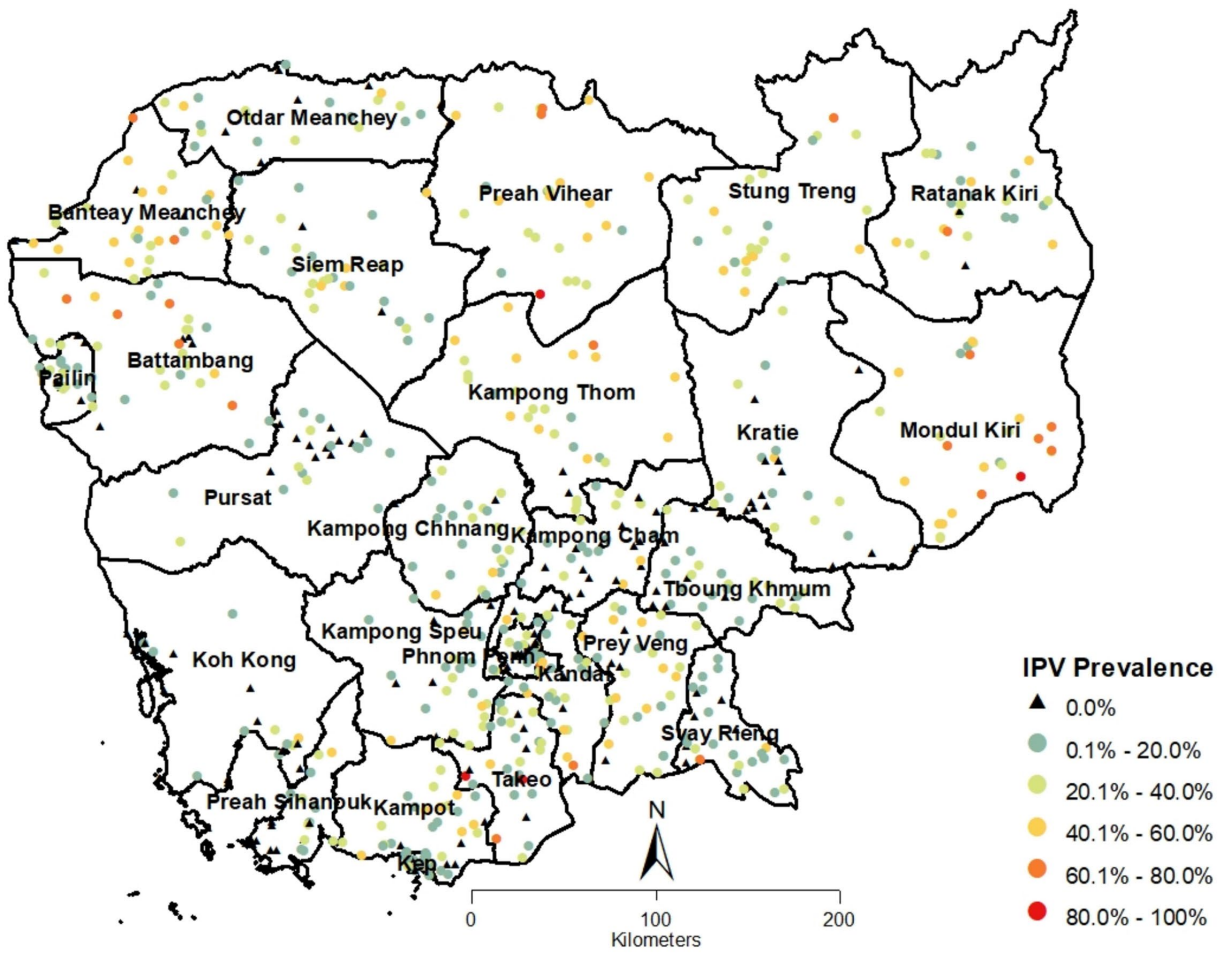
Spatial distribution of cluster points with proportions of IPV in Cambodia.

The proportion of intimate partner violence in Cambodia was 20.70% (95% CI: 19.25–22.23%) among women aged 15–49. This includes violence committed by their current or most recent husband for ever-married women, or by their current or most recent intimate partner for never-married women. At the EA level, IPV proportions ranged from 0 to 100%, as shown in Figure 1, which highlights the EA locations and their corresponding IPV proportions across different provinces. Northern half of the country had more EAs with higher IPV proportions, while southern half had more EAs with lower IPV proportions.

The spatial analysis revealed that IPV prevalence in Cambodia is not randomly distributed but exhibits clear spatial clustering. The Global Moran’s I statistic (Moran’s I Index: 0.223079; Z Score: 16.914397; *p*-value: <0.0001) indicated statistically highly significant positive spatial autocorrelation with the conceptualization of spatial relationship as ‘Zone of Indifference’ using Euclidean distance method. This finding suggests that areas with high IPV prevalence tend to be geographically close, as do areas with low prevalence. The results using ‘Inverse Distance’ method was also statistically highly significant. The highly significant *p*-values observed in the results indicated robust spatial autocorrelation, regardless of the conceptualization method used. These consistent results reinforce the robustness of the findings.

Further analysis using the Getis-Ord Gi* statistic was performed to identify significant hot and cold spots of IPV prevalence. The hot spot analysis highlighted areas with significantly high or low levels of IPV, providing crucial insights into geographic patterns that warrant targeted intervention. Using the Getis-Ord Gi* statistic, significant hot and cold spots of IPV prevalence were identified, offering key insights for targeted interventions.

**Figure 2:**
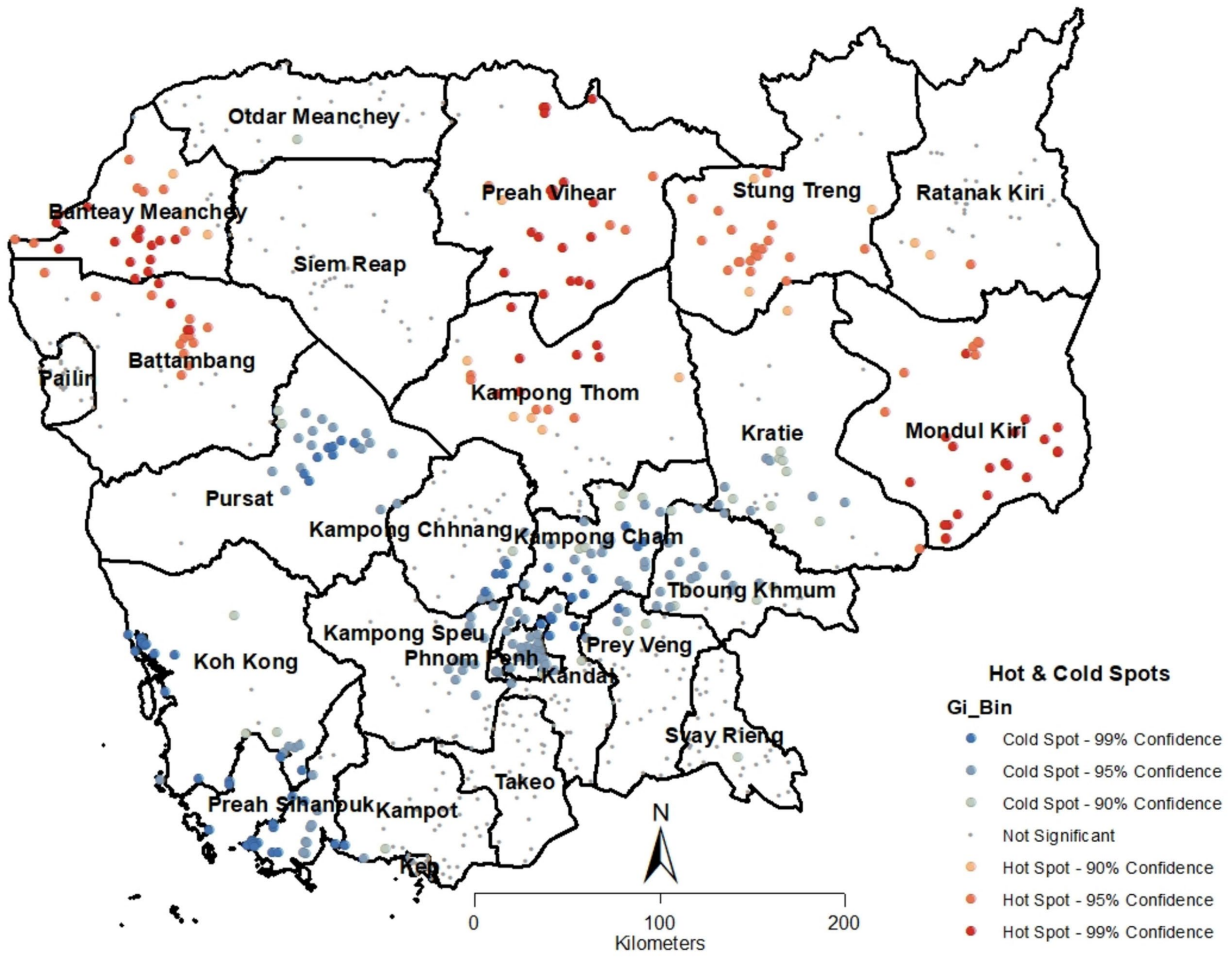
Results of intimate partner violence hot-spot analysis based on Getis Ord (Gi*) statistic in Cambodia.

Figure 2 shows the results of hot spot analysis based on Getis Ord (Gi*) statistic, which bore out the results in Figure 1. Hot spots were exclusively present in the northern half of the country with provinces of Mondal Kiri, Stung Treng, Preach Vihear, Banteay Mearchey, Ratanak Kiri and Battambang showing pronounced hot spots. While southern part of the country was replete with cold spots, with pronounced concentrations in the provinces of Phnom Phenh, Koh Kong, Kampang Cham, Pursat, Preah Sihanouk, and Karatie.

**Figure 3:**
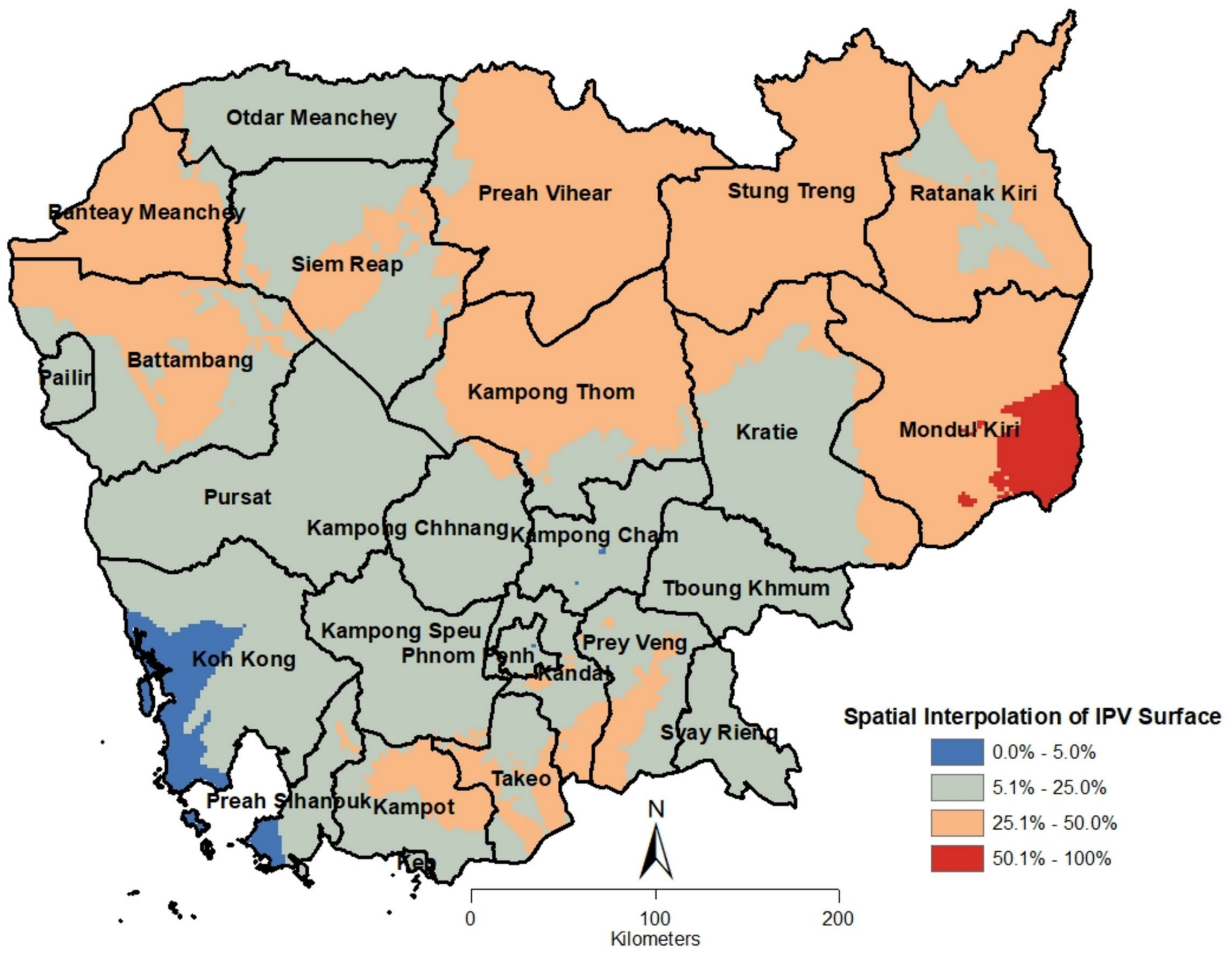
Cambodia intimate partner violence kriged surface.

The interpolated surface of IPV proportions, based on ordinary kriging, is displayed in Figure 3. These results generally align with and complement the findings from the previous two analyses. Large areas in the northern half of the country, as well as smaller regions in the southern provinces of Kampot, Takeo, Kandal, and Prey Veng, showed IPV prevalence proportions ranging from greater than 25 to 50%. The southeastern part of Mondul Kiri province exhibited the highest IPV proportions, ranging from over 50 to 100%. In contrast, the lower half of the country had comparatively lower IPV prevalence, with proportions between greater than 5 and 25%. The western parts of Koh Kong and Preah Sihanouk provinces had the lowest IPV proportions, ranging from 0 to 5%. Across the rest of the country, IPV prevalence ranged from 5.1 to 25%. However, it’s important to note that these IPV prevalence estimates from the kriged surface are approximations.

## Discussion

This is the first study to present a spatial analysis of intimate partner violence (IPV) in Cambodia, with a focus on identifying clusters, hot and cold spots of prevalence, and generated an interpolated surface to visualize the spatial variation in IPV. The findings revealed distinct geographic clustering of IPV, significant hotspots in certain regions; distinct distribution and disparities in IPV prevalence across the country. The spatial analysis offers a crucial avenue for understanding the geographic distribution and disparities of IPV, helping to inform targeted interventions aimed at mitigating its impact. The spatial clustering of IPV, as demonstrated by the significant Moran’s I value, suggests that IPV is not randomly distributed across Cambodia but rather forms spatial clusters. This clustering highlights the geographic concentration of risk factors that contribute to IPV, indicating that women residing in certain areas are more susceptible to experiencing violence compared to others. The presence of both hotspots and cold spots of IPV prevalence further emphasizes the geographic disparities in the burden of IPV, influenced by a range of socio-economic, cultural, geographical, and environmental factors. The interpolated surface provided a visual representation of the variations in IPV prevalence in the country.

In general, the northern provinces of the country were abundant in hotspots, indicating a statistically significant higher prevalence of intimate partner violence (IPV). In contrast, the central and southern provinces were characterized by cold spots, which demonstrated a statistically lower prevalence of IPV. However, there were notable exceptions, including several provinces that had very few or no statistically significant hotspots. These provinces included Ratanak Kiri, Otdar Meanchey, Siemreap, Kampong Chhnang, Prey Veng, Takeo, and Pailin.

Research from various countries has highlighted significant geographical disparities, both across national territories and within administrative subdivisions. National and subnational variations in IPV prevalence have been documented in several studies (2–5, 9).

The findings from this spatial analysis have important implications for public health policy, judicial reforms, and targeted interventions aimed at reducing IPV in Cambodia. The identification of specific hotspots of IPV prevalence provides an opportunity for targeted intervention in the regions most affected by IPV. Policymakers should prioritize resource allocation to these areas, ensuring that women in high-risk regions have access to essential services such as shelters, legal aid, and counseling services. Community-based programs focusing on economic empowerment, education, and awareness campaigns to challenge harmful gender norms are also essential. Such interventions should involve community leaders and local organizations to ensure cultural relevance and sustainability.

The interpolated surface of IPV prevalence provides a valuable tool for geographic targeting of resources and interventions. By visualizing variations in IPV prevalence across regions, policymakers can identify not only the areas with the highest need but also neighboring areas that may be at risk. Such data-driven approaches ensure that interventions are directed where they are most needed, optimizing the use of limited resources.

This study represents one of the first attempts to use spatial analysis to explore IPV prevalence in Cambodia, offering valuable insights for targeted interventions. The use of Geographic Information Systems (GIS) techniques allowed for a detailed visualization of spatial patterns, providing a nuanced understanding of how IPV risk varies across regions.

The primary strength of this study lies in its use of nationally representative data, ensuring broad applicability and reliability of the findings. However, like any study, it is not without limitations. The data utilized in this study were derived from the Demographic and Health Survey, a widely recognized and robust source of secondary data. While self-reported data on IPV may indeed be affected by stigma and reporting bias, the DHS employs rigorous protocols, including interviewer training and confidentiality assurances, to minimize these biases. However, it is acknowledged that some underreporting may still occur. Future research could complement DHS data with qualitative approaches or community-based surveys to enhance data reliability. Secondly, the GPS displacement applied by DHS for privacy protection, introduces spatial inaccuracy. Nonetheless, the magnitude of this impact is limited for the objectives of this study, which focused on broader spatial patterns, clustering, and hotspot identification rather than highly localized effects. Thirdly, this study utilized cross-sectional DHS data, which limits the ability to infer causality. As the dataset is secondary and inherently cross-sectional, causation was not within the scope of this analysis. Future studies could build on these findings by incorporating longitudinal designs or triangulating with qualitative data to better understand the underlying factors associated with IPV. Fourthly, it is acknowledged that IPV clusters and hotspots identified in this study may evolve over time due to social, economic, and cultural changes. This study provides a foundational analysis of IPV spatial patterns, which can be complemented by longitudinal research to assess temporal stability and dynamics. Such studies would provide further insights into the shifting nature of IPV and its spatial determinants. Finally, while previous studies have explored economic, cultural, and social factors influencing IPV, this study specifically aimed to examine the spatial distribution of IPV and identify clusters, hot and cold spots, and apply Kriging analysis. Incorporating broader socio-economic factors in spatial modeling was beyond the scope of the study’s objectives but represents an important area for future research.

Future studies investigating the spatial distribution of IPV in Cambodia should consider incorporating Geographically Weighted Regression (GWR) and spatial Bayesian analysis for a more nuanced and robust analysis. GWR is a spatial technique that examines relationships between variables at local levels, uncovering geographic variations often missed by global models. Spatial Bayesian analysis, on the other hand, leverages prior information and probabilistic modeling to account for spatial dependencies and uncertainty, providing deeper insights into IPV patterns. Together, these approaches can offer a comprehensive understanding of IPV correlates and inform targeted, context-specific interventions.

This study provides a valuable foundation for designing targeted interventions to address IPV in identified hotspots, emphasizing the importance of geographic disparities in understanding and addressing IPV. While feasibility studies on the effectiveness and cost of such interventions fall outside the scope of this analysis, future research should build on these findings to pilot and evaluate practical strategies. By highlighting the role of place in IPV prevalence, this study offers insights that can guide policymakers, service providers, and community organizations in developing effective, location-specific interventions to reduce IPV and improve the lives of women across Cambodia.

## Data Availability

The data analyzed in this study is subject to the following licenses/restrictions: the ethical approval for the CDHS2021-22 was granted by the Cambodian National Institute of Statistics and the Ministry of Health, in addition to the ICF Institutional Review Board. The approval for the conduct of secondary analysis of fully anonymized CDHS 2021-22 survey and spatial data was granted to the author by Measure DHS using the online request form. Requests to access these datasets must be submitted to the DHS Program using the online portal available at Measure DHS website: www.measuredhs.com.

## Footnotes

^1^www.measuredhs.com
